# The Association between GABA-Modulators and *Clostridium difficile* Infection – A Matched Retrospective Case-Control Study

**DOI:** 10.1371/journal.pone.0169386

**Published:** 2017-01-06

**Authors:** Jonathan Ström, Johan Tham, Fredrik Månsson, Jonas Ahl, Tor C. Savidge, Sara M. Dann, Fredrik Resman

**Affiliations:** 1 Infectious Diseases Unit, Department of Translational Medicine, Lund University, Malmö, Sweden; 2 Department of Pathology and Immunology, Baylor College of Medicine, Houston, Texas, United States of America; 3 Texas Children’s Microbiome Center, Texas Children’s Hospital, Houston, Texas, United States of America; 4 Department of Internal Medicine, University of Texas Medical Branch, Galveston, Texas, United States of America; Cleveland Clinic, UNITED STATES

## Abstract

**Objective:**

Recently, metabolomics studies have suggested that the neurotransmitter γ-amino butyric acid (GABA) may modulate *C*. *difficile* infection (CDI) pathogenesis. In the present study, we investigated the association between GABA-modulating pharmaceuticals and CDI development.

**Methods:**

In July-December 2013, we performed a matched, retrospective case-control study in Skåne county, Sweden, to assess the association between the use of GABA-modulators (defined as regular use of at least one of the following: zolpidem, zopiclone, benzodiazepines, gabapentin, pregabalin or baclofen) and CDI. Multivariate regression models, adjusted for known risk factors for CDI, were fitted to assess the associations and a propensity score-adjusted analysis was performed.

**Results:**

The study included 292 cases and 292 matched controls. In a multivariate regression model only recent antibiotic use (clindamycin, cephalosporins and fluoroquinolones) and nursing home residency was significantly associated with CDI. The regular use of any GABA-modulator was not associated with CDI (OR = 1.07, 95%CI 0.69–1.66, *p* = 0.76). The association between regular use of the selective GABA-agonist zolpidem and CDI trended towards significance (OR = 2.31, 95%CI 0.91–5.86, *p* = 0.078). These associations remained when only cases treated with antibiotics were included. Corresponding findings for zolpidem was observed in a propensity-score adjusted analysis (OR = 2.52, 95% CI 0.91–6.97, *p* = 0.075). Severe initial CDI was significantly associated with CDI recurrence (OR = 3.77, 95% CU 1.20–11.86, *p* = 0.023).

**Conclusion:**

This study did not identify a general association between GABA-modulators and CDI. A trend towards a significant association between zolpidem and CDI was observed, an association that should be re-assessed in a study appropriately powered for this particular hypothesis.

## Introduction

*Clostridium difficile*-infection (CDI) is the most common nosocomial infection that requires inpatient care in adults, and the incidence is increasing at an alarming rate [1,2]. In US hospitals, the rate of CDI listed as a diagnosis increased from 3.8 to 8.7 per 1,000 discharges between the years 2000 and 2008 [2]. In Sweden, CDI incidence is estimated at 80 cases per 100,000 individuals per year [3]. CDI is associated with a significant attributed mortality. Despite effective treatment options, the case fatality rate of CDI increased from 5–7% to around 10% in the 2000’s [4,5]. This may in part be due to the epidemic spread of more virulent strains, such as ribotype 027 [6,7].

Two distinct steps are required for CDI to occur, bacterial transmission and alteration of the gastrointestinal flora [8]. The use of antibiotics that disrupt the gastrointestinal flora is the most important risk factor for CDI development, with the vast majority of CDI cases preceded by antibiotic treatment [2]. Other risk factors include those mainly related to the risk of bacterial transmission such as recent hospitalization and nursing home residency, and those related to vulnerability such as old age and frailty [9]. Based on these risk factors, CDI is considered primarily a health-care associated infection [10]. In addition to antibiotics, several other pharmaceutical agents have been shown to affect the risk of CDI-development in various ways, including proton pump inhibitors (PPI), antidepressants, statins and corticosteroids [11–15].

GABA, γ-aminobutyric acid, is a major neurotransmitter in the central nervous system, but also has modulatory effects on enteric neurons and immune cells [16,17]. GABA mediates its action through several isoforms of the GABA-receptors that are differentially expressed across cell types and tissues. Receptor activation induces immunosuppression, which has led many to evaluate its potential as a therapeutic target of neuroinflammatory diseases [16,18]. Recently, global metabolomic profiling of stool samples from CDI patients, as well as animal models, revealed increased GABA levels are associated with CDI and are a strong predictor for disease recurrence, suggesting that the neurotransmitter may have a role in disease susceptibility and/or pathogenesis [19].

Several widely prescribed medications with sedative and musculoskeletal relaxant properties elicit their actions by acting as ligands or modulators of GABA receptors. Gabapentin and pregabalin, used to treat neuropathic pain and seizures, are structural analogues of GABA that modulate GABAergic responses independently of receptor binding; whereas baclofen, which is used primarily as a muscle relaxant, is a selective GABA_B_-receptor agonist. Benzodiazepines and barbiturates, commonly used to treat anxiety and sleeping disorders, potentiate the effect of GABA by binding near or to specific sites within the GABA_A_-receptor, resulting in the enhancement of receptor transmission. Although most GABA ligands are non-specific and bind several receptor isoforms, some medications, such as the hypnotic agent zolpidem, are selective for certain GABA_A_-receptor isoforms [20].

The purpose of this study was to investigate the association between GABA-modulating drug use and CDI through a retrospective matched case-control design.

## Materials and Methods

### Study setting

The study was performed in Skåne county, Sweden (population approx. 1,200,000). Skåne county is served by one central public laboratory for clinical microbiology where all microbiology diagnostics is performed. No private alternatives are available. Furthermore, nine out of ten hospitals in Skåne county, including the six largest hospitals, have a common electronic medical records system. This makes the area suitable for comprehensive epidemiological investigations of hospital-related infections.

### Study population

All patients above 18 years of age in Skåne county who had been tested positive, at a hospital, for *Clostridium difficile* (CD) during the period July 1^st^ to December 31^st^ 2013 and at the time of the test had an acute-onset diarrhoeal episode were included as potential cases and were approached for study inclusion. Information on the exact number of loose stools per day was not possible to extract from the medical records. Potential cases in which reliable information on predictor variables could not be obtained from the medical records were excluded. Patients with repeat positive cultures were included only once. For each case, a randomly selected age- and gender-matched individual sampled at a hospital for *C*. *difficile* in the period July 1st—December 31^st^ but tested negative was approached. Age was matched to the same five-year range. Potential controls in which reliable information on predictor variables could not be obtained from the medical records were excluded. The final control group consisted of a case-corresponding number (1:1) of age and gender-matched patients.

### Culture and testing

Each sample was cultured to detect CD. The presence of toxins were tested with Premier ^™^ Toxins A & B (Meridian Laboratory Corp. Charlotte, NC, USA), based on ELISA technique.

### Descriptive variables

The following descriptive variables were collected from all cases and controls: *age*, *gender*, *current type of housing* (own home or nursing home), *recent hospitalization* (defined as hospitalization for longer than 24 hours within two months prior to sampling), *comorbidities* (the comorbidities included in the Charlson’s comorbidity index [21]) *immunodeficiency* (including primary immunodeficiency, neutropenia or chemotherapy given within one month but not biologics), *inflammatory bowel disease*, *recent history of depression*.

### Predictor variables

Information on the following known or suggested predictor variables (risk factors) for CDI were also collected from all cases and controls: *antibiotic use during the two months prior to the C*. *diff test* as well as *ongoing/recent use of the following pharmaceuticals*: proton pump inhibitors, statins, corticosteroids, SSRI, SNRI, mirtazapine as well as GABA-modulators. Use of the following GABA-modulators was recorded; zolpidem, zopiclone, benzodiazepines, gabapentin, pregabalin and baclofen.

### Outcome variables

Information on 28-day mortality was collected from all cases and controls. For each case, severity of the infection, CDI recurrence (an episode that occurred within eight weeks of the onset of a previous episode [22]) as well as the antibiotic treatment choice of the initial period was recorded.

### Data collection

Information on all descriptive variables and predictor variables was collected from medical records. All information on descriptive variables correlated to the time prior to (comorbidities) and at the time of the sampling (all other variables). All information on predictor variables correlated to the time of the sampling, and for antibiotics, to the two months prior to the sampling. Since only patients sampled at hospital were included, comprehensive information on comorbities and current medication was the rule. For each medication except for GABA-modulators and antibiotics, regular/daily use was recorded. For each GABA-modulator, regularity of use was recorded. Five days per week or more was considered as regular use. Individuals with sporadic use were not excluded from the respective cohorts. For antibiotics, the exact number of days of treatment was not recorded.

All information on outcome variables correlated to the time after the sampling. 28-day mortality was assessed through the electronic administrative records (Pasis). The severity of infection was categorized into mild infection, severe infection and pseudomembranous colitis/death according to the IDSA/SHEA guidelines [23].

### Data analysis

The Wilcoxon rank-sum test was used to compare descriptive variables between cases and controls. For non-continuous descriptive variables, chi-square was used to assess differences between groups. Univariate logistic regressions were performed for all predictor variables, and odds ratios (OR) (including 95% confidence intervals) were calculated.

The association between regular use of GABA-modulators and CDI was assessed using multivariate logistic regression models. Two models assessing the association between the use of any GABA modulator, defined as regular use of at least one of the included GABA-modulators (zolpidem, zopiclone, benzodiazepines, gabapentin, pregabalin and baclofen), and CDI were fitted; with CDI and with CDI that needed antibiotic treatment. The second analysis (for only individuals that received antibiotic treatment for CDI) was performed in order to separate more severe CDI cases from the group with mild disease. This was performed since we could not safely assess the number of stools per day of all cases from a retrospective analysis of the medical records (and thus could be sure that each case met the standard case definition of CDI). Since the use of zolpidem was significantly associated with CDI in a univariate model, the multivariate CDI models above were also tested for the association with regular use of zolpidem. A range of plausible effect modifiers for zolpidem and interaction terms between covariates, selected based on subject matter knowledge was assessed (S1 Table). The multivariate model assessing the association between zolpidem use and CDI was stratified on individuals with and without history of depression, since history of depression was suggested as an effect modifier in the association between zolpidem use and CDI (S1 Table). The association between predictor variables and recurrent CDI was assessed in a separate model, comparing recurrent and non-recurrent CDI, and this time also including initial treatment and CDI severity as predictor variables.

Age and gender were included and kept in all final multivariate models regardless of *p*-value. The full models were fitted using the purposeful selection algorithm, with limits of *p*-values at 0.1 and predictor changes at 20% [24]. The ‘one in ten’ rule (a maximum of one predictor variable per ten events/cases to reduce the risk of overfitting) [25] was used to limit the number of predictors in the final model, and in the model for recurrent CDI, where the number of cases were limited, only the most significant covariates were kept (according to *p*-value assessment). Finally, all models were tested for goodness-of-fit using the Hosmer-Lemeshow test and assessed for discriminatory accuracy calculated as the area under the receiving operator curve (AU-ROC).

To further assess the association between zolpidem use and CDI, a propensity-score adjusted analysis of the association was performed. The propensity score was calculated using regular zolpidem use as the outcome variable in a multivariate regression including all potential confounders and reasonable interaction variables (based on subject matter experience) measured in the study (S2 Table). The propensity score was used as a covariate in a regression analysis assessing the association between CDI and regular use of zolpidem.

All analyses were performed using STATA14^®^ (Statacorp, College Station, Texas, USA).

### Ethical considerations

Permission to perform this study according to the study design was granted by the regional ethical review board in Lund, Sweden (2013/845). Consent was given through opt-out letters sent to all potential cases and controls in the study. This consent procedure was approved by the regional ethical review board. All data were analyzed anonymously.

## Results

### Study population

The total number of cases considered for inclusion was 313. Four patients denied participation, and in 17 cases (5.5%) the medical records lacked sufficient information on descriptive and/or predictor variables, leaving 292 patients for analysis. In the control group, 309 patients were approached before 292 controls had been identified. Three patients denied participation, and in 14 controls (4.6%) the medical records lacked sufficient information on descriptive and/or predictor variables.

### Baseline comparison between the case and control group

All descriptive variables were compared between cases and controls (Table 1). Age and gender did not differ between groups due to matching of controls. No significant difference in the Charlson’s comorbidity index or for any individual condition within the index was identified between groups. No difference in the proportion of individuals with immunodeficiency was identified between the groups. There were significantly more patients with ulcerative colitis among controls, likely indicating that these patients are often sampled and tested negative for CDI. Unsurprisingly, nursing home residency and recent hospitalization (within the two months prior to sampling) were significantly more common among cases (Table 1). More surprisingly, recent history of depression was recorded more often among controls.

**Table 1:** The distribution of descriptive variables in the case and control groups, respectively.

	CDI-cases cohort (*n*, % unless stated)	Control cohort (*n*, % unless stated)	*P*-value
**Total cases**	292	292	
**Age (median, IQR)**	74 (63.5–82)	74 (62–83)	0.83
**Gender (% women)**	53.4	51.7	0.68
**Nursing home residency**	45 (15.4%)	26 (8.6%)	0.008*
**Recent hospitalization**	155 (53.1%)	103 (35.3%)	<0.001*
**Peripheral vascular disease**	28 (9.6%)	20 (6.9%)	0.23
**History of Myocardial infarction**	48 (16.4%)	54 (18.5%)	0.51
**Congestive Heart Failure**	54 (18.5%)	39 (13.4%)	0.09
**Cerebrovascular disease**	48 (14.4%)	42 (16.4%)	0.49
**Chronic mild liver disease**	3 (1.0%)	1 (0.3%)	0.32
**Dementia**	16 (5.5%)	13 (4.5%)	0.57
**Chronic pulmonary disease**	39 (13.4%)	35 (12.0%)	0.62
**Rheumatologic disease**	30 (10.3%)	32 (11.0%)	0.79
**Peptic ulcer**	22 (7.5%)	35 (12.0%)	0.07
**Diabetes without organ failure**	33 (11.3%)	48 (16.4%)	0.09
**Non-metastatic solid tumor**	43 (14.7%)	47 (16.1%)	0.65
**Hemiplegia**	6 (2.1%)	9 (3.1%)	0.43
**Leukemia/Lymphoma**	17 (5.8%)	13 (4.5%)	0.46
**Diabetes with organ failure**	26 (8.9%)	26 (8.9%)	1
**Chronic kidney disease**	45 (15.4%)	43 (14.7%)	0.59
**Moderate/severe liver disease**	9 (3.1%)	9 (3.1%)	1
**AIDS**	0	0	-
**Metastatic malignant tumor**	16 (5.5%)	20 (6.9%)	0.49
**Charlson’s Comorbidity index (median, IQR)**	2 (1–4)	2 (1–4)	0.81
**Immunodeficiency**	46 (15.8%)	44 (15.1%)	0.82
**Crohn’s disease**	10 (3.4%)	16 (5.5%)	0.23
**Ulcerative colitis**	10 (3.4%)	23 (7.9%)	0.02*
**History of depression**	45 (15.4%)	66 (22.6%)	0.03*

*Statistically significant difference

### Univariate analyses of predictor variables

In univariate analyses, the use of clindamycin, cephalosporins, fluoroquinolones or Co-trimoxazole in the two months prior to sampling was significantly associated with CDI (Table 2). In patients that were given penicillin alone, no increased risk was identified. Among non-antibiotic pharmaceuticals, no association was seen between the regular use of GABA-modulators (from the included drugs) and CDI when grouped together, but a significant association between regular use of zolpidem and CDI was identified. Corticosteroid treatment was significantly associated with CDI development. Neither use of PPI, statins or antidepressants was associated with CDI in our cohort (Table 2). The assessment of potential effect modifiers and interaction terms is displayed in S1 Table.

**Table 2:** Univariate associations between collected predictor variables and *Clostridium difficile*-infection.

Medication	Cases with treatment (*n*)	Controls with treatment (*n*)	Odds ratio (95% Confidence interval)	*P*-Value
**Penicillins**	126	93	1.62 (1.15–2.28)	0.005*
**Cephalosporins**	110	45	3.31 (2.23–4.93)	<0.001*
**Clindamycin**	41	7	6.65 (2.93–15.1)	<0.001*
**Carbepenems**	25	21	1.21 (0.66–2.21)	0.54
**Fluoroquinolones**	52	18	3.30 (1.88–5.79)	<0.001*
**Tetracycline**	3	5	0.60 (0.14–2.52)	0.48
**Co-trimoxazole**	28	11	2.71 (1.32–5.55)	0.006*
**Penicillin only**^1^	45	60	0.70 (0.46–1.08)	0.11
**SSRI**	36	46	0.75 (0.47–1.20)	0.235
**Mirtazapin**	22	15	1.50 (0.76–2.96)	0.24
**PPI**	133	126	1.10 (0.79–1.53)	0.56
**Statin**	90	86	1.06 (0.75–1.52)	0.72
**Corticosteroids**	91	69	1.46 (1.01–2.11)	0.042*
**Any GABA-modulator**^2^	79	68	1.22 (0.84–1.78)	0.30
**Benzodiazepines**^2^	23	29	0.78 (0.44–1.38)	0.38
**GABA-analogs**^2^	17	17	1 (0.5–2.0)	1
**Baclofen**^2^	5	3	1.68 (0.40–7.09)	0.48
**Zopiclone**^2^	36	37	0.97 (0.59–1.58)	0.90
**Zolpidem**^2^	22	8	2.89 (1.27–6.60)	0.012*

^1^ All patients that were administered another antibiotics as well removed

^2^ Only regular use (5 or more days per week) was considered

* Statistically significant association

### Multivariate regressions for *Clostridium difficile* development

Multivariate models were fitted assessing the association between regular use of any included GABA-modulator and CDI. Two outcomes were assessed separately; patients with CDI (*n* = 292) and patients with CDI that received antibiotic treatment (*n* = 226). The analyses of the associations between any GABA-modulator and CDI are presented in Table 3. In summary, there was no association between regular use of any included GABA-modulator and CDI or CDI that needed antibiotic treatment. Four covariates were significantly associated with CDI in the models: nursing home residency, recent cephalosporin use, recent clindamycin use and recent fluoroquinolone use. The odds ratios of these predictors increased when only cases treated with antibiotics were included in the analysis. History of depression was significantly associated with controls. When history of depression was removed from the analysis, SSRI treatment became significantly associated with controls without affecting other inferences. The Hosmer-Lemeshow test indicated acceptable goodness of fit for the models.

**Table 3:** Multivariate logistic regression models determining associations between the regular use of any GABA-modulator and CDI as well as CDI treated with antibiotics.

Variable	Analysis with all CDI cases		Analysis with CDI cases treated with antibiotics only	
	Odds ratio (95% CI)	*p*-value	Odds ratio (95% CI)	*p*-value
**Use of any GABA-modulator**	1.07 (0.69–1.66)	0.762	1.07 (0.66–1.74)	0.773
**Gender (male is baseline)**	1.07 (0.69–1.66)	0.485	1.37 (0.91–2.06)	0.136
**Age**	0.99 (0.98–1.00)	0.062	0.99 (0.98–1.00)	0.080
**Recent hospitalization**	1.41 (0.95–2.09)	0.085	-^1^	-
**Nursing home residency**	4.1 (1.91–8.80)	<0.001	5.86 (2.61–13.18)	<0.001
**Depression**	0.45 (0.27–0.74)	0.002	0.46 (0.27–0.80)	0.005
**Cefalosporines**	5.05 (3.06–8.33)	<0.001	6.13 (3.56–10.56)	<0.001
**Clindamycin**	8.2 (4.84–44.15)	<0.001	20.4 (6.55–63.55)	<0.001
**Fluoroquinolones**	6.09 (2.87–12.93)	<0.001	8.35 (3.77–18.53)	<0.001
**Co-trimoxazole**	2.11 (0.96–4.62)	0.063	2.68 (1.17–6.13)	0.019
**Nursing home residency# cephalosporines**	-	0.015	-	0.004
**Nursing home residency# clindamycin**	-	0.002	-	0.001
**Cephalosporines#fluoroquinolones**	-	0.002	-	0.002
**AU-ROC of the model**	0.75		0.77	

^1^ The *p*-value was >0.1 in the final model, and thus the predictor was removed.

The association between regular zolpidem use and CDI, as well as CDI that received antibiotic treatment, was assessed separately. Since history of depression was identified as a possible effect modifier of the association between zolpidem and CDI (S1 Table), which was true also for CDI that needed treatment (data not shown), three analyses were performed on each association. One in only individuals with depression, one in only individuals without depression and one without the depression predictor in the analysis. The analyses of the association without history of depression included as a covariate are shown in Table 4. The associations between zolpidem and CDI (OR = 2.31, 95% CI 0.91–5.86, *p* = 0.078) and CDI that needed treatment (OR = 2.36, 95% CI 0.88–6.38, *p* = 0.089) trended towards significance in these models. In a model including only patients with no history of depression (*n* = 473, 81% of the population), regular zolpidem use was significantly associated with CDI (OR 5.58 95% CI 1.17–26.60 *p* = 0.031, model AU-ROC = 0.76) and with CDI that needed treatment (OR 6.57, 95% CI 1.33–32.50, *p* = 0.021, model AUC-ROC 0.79). In a model including only patients with history of depression (*n* = 111, 19% of the population) regular zolpidem use was not associated with CDI (OR 1.18 95% CI 0.23–5.91 *p* = 0.842, model AU-ROC = 0.73) or CDI that needed treatment (OR 0.69, 95% CI 0.06–7.47, *p* = 0.760, model AU-ROC = 0.78). The Hosmer-Lemeshow test for goodness of fit indicated that all models had acceptable fit.

**Table 4:** Final multivariate logistic regression models determining the associations between the regular use of zolpidem and CDI as well as CDI treated with antibiotics.

Variable	Analysis with all CDI cases		Analysis with CDI cases treated with antibiotics only	
	Odds ratio (95% CI)	*p*-value	Odd ratio (95% CI)	*p*-value
**Regular use of zolpidem**	2.31 (0.91–5.86)	0.078	2.36 (0.88–6.38)	0.089
**Gender (male is baseline)**	1.12 (0.78–1.61)	0.541	1.36 (0.91–2.05)	0.138
**Age**	0.99 (0.98–1.00)	0.109	0.99 (0.98–1.00)	0.101
**Nursing home residency**	4.1 (1.93–8.77)	<0.001	5.66 (2.52–12.71)	<0.001
**SSRI**	0.56 (0.32–0.96)	0.036	0.52 (0.28–0.94)	0.032
**Cephalosporines**	5.16 (3.14–8.49)	<0.001	5.88 (3.41–10.14)	<0.001
**Clindamycin**	13.6 (4.57–40.37)	<0.001	18.5 (5.98–56.96)	<0.001
**Fluoroquinolones**	6.24 (2.99–13.02)	<0.001	7.67 (3.49–16.85)	<0.001
**Co-trimoxazole**	2.52 (1.15–5.52)	0.021	2.68 (1.17–6.13)	0.019
**Nursing home residency# cephalosporines**	-	0.016	-	0.006
**Nursing home residency# clindamycin**	-	0.003	-	0.001
**Cephalosporines#fluoroquinolones**	-	0.002	-	0.002
**AU-ROC of the model**	0.74		0.77	

### Propensity score regression

A propensity score for the use of zolpidem was created, using potential confounders and interaction variables (S2 Table), added as a covariate in a regression model evaluating the association between CDI development and regular zolpidem use. Again, the association between regular zolpidem use and CDI development trended towards statistical significance (OR = 2.52, 95% CI 0.91–6.97, *p* = 0.075).

### Mortality and disease severity

The 28-day mortality did not differ between cases and controls as 43 (14.7%) individuals died within 28 days among cases and 37 (12.6%) individuals among controls, *p* = 0.47. According to the severity classification only 17 had severe CDI (6%) and three had pseudomembranous colitis (1%).

### Treatment and recurrence

A total of 69% of patients were treated with metronidazole and 20% of patients were treated with vancomycin (some were treated with both, in combination or in sequence). Two patients received fidaxomicin and three received fecal microbiota transplant. Disease recurrence occurred in 35 (17%) patients who received metronidazole alone, and five patients who received vancomycin (15%). A regression model was fitted for the risk of recurrence. The best predictors of recurrence were severe primary CDI, leukemia and solid tumor with metastasis. However, the predictors in this analysis did not predict recurrence well, and the final model (Table 5) had poor discriminatory accuracy, AU-ROC (0.60). There was no evidence of an association between the use of any GABA-modulator and CDI recurrence.

**Table 5:** Final, adjusted regression model for the risk of recurrent CDI.

Variable	Odds Ratio (95%Confidence Interval)	*p*-value
**Use of any GABA-modulator**	0.88 (0.47–1.65)	0.699
**Severe initial CDI**	3.76 (1.31–10.79)	0.014
**Solid tumor with metastasis**	1.18 (1.00–1.41)	0.050
**Leukemia**	2.03 (1.08–3.83)	0.029

## Discussion

In this matched retrospective case-control study we did not identify an association between the regular use of a GABA-modulator and CDI. This does not exclude the possibility that individual GABA-modulators may be associated with risk, and the association between regular use of zolpidem, a selective GABA-_A_ agonist, and CDI trended towards statistical significance. The study also re-confirms the antibiotics mainly associated with CDI development: clindamycin, fluoroquinolones and 3^rd^ generation cephalosporins. Nursing home residency was independently associated with CDI in the study. Identifying risk factors associated with the risk of recurrent CDI was more challenging, but the severity of the initial infection seemed to be associated with the risk of recurrence.

Our study, while not designed (and thereby powered) to fully assess association between CDI and individual GABA-modulators, identified a trend towards a significant association between CDI and regular use of zolpidem. In a prospective case-controlled study conducted in the Texas Medical Center in Houston, Texas, a significant association between zolpidem use and CDI was measured, with an increased relative risk of 4.8 [19,26]. Together, these data identify zolpidem as a potential new risk factor for CDI development. Zolpidem is an allosteric modulator of GABA_A_ receptors and enhances signaling transmission by GABA. In bacterial pneumonia, activation of GABA_A_ receptors by benzodiazepine use impairs innate immune function resulting in higher bacterial burden and mortality. In comparison to other GABA-modulating agents, zolpidem is selective for certain GABA_A_ receptor isoforms, particularly those expressing the alpha1 subunit that is expressed on many cell types throughout the intestines, including leukocytes. In a recent study, patients using zolpidem had an increased risk of developing enteritis and increased general susceptibility to infection [27]. Although the mechanisms of action underlying the effects of zolpidem on CDI susceptibility are unknown, there is evidence to suggest zolpidem modulates protective innate and adaptive immune defenses, and studies are being conducted to elucidate the impact of zolpidem on host responses to CDI.

In the analysis, an effect modification on the association between zolpidem and CDI was observed. When patients with a recent history of depression were excluded from the analysis, regular zolpidem use was identified as a statistically significant risk factor for CDI; whereas zolpidem use had no effect on individuals diagnosed with depression. In our study, history of depression was associated with controls, contrary to findings from a prior study [13]. The finding is interesting, but it should be noted that even though careful assessment of variables was performed, history of depression is a factor prone to non-differential misclassification. Interestingly, when an analysis was performed following exclusion of the depression covariate, SSRI treatment was significantly associated with controls in a fully adjusted model. Use of SNRI was also collected, but very few individuals in the study used SNRI, and it did not affect the results.

Not surprisingly, clindamycin, fluoroquinolones, 3^rd^ generation cephalosporins were all associated with an increased risk of CDI. Somewhat less predicted, penicillin treatment was not associated with disease development. The most likely reason for this is that penicillin does not disrupt the intestinal flora to the same extent as ‘high-risk’ antibiotics, but it is interesting to note that penicillin appears to be able to affect GABA_A_-signaling through the blocking of receptor ion channels [28]. Contrary to previous research, no association with risk of developing CDI was identified for the use of neither PPI nor statins [11,12,14].

This study has strengths, limitations and issues where alternative strategies could be discussed. The strengths of the study include the detailed patient-level information that was possible to retrieve from medical records. Another strength is the careful matching of controls to cases, and the thorough analysis of the material. The limitations include that individual ribotypes of *Clostridium difficile*, a factor known to affect the risk of CDI as well as severe CDI, could not be identified from the material and that dosing regimens and lengths-of-treatment of antibiotics were not collected. The fact that cases and controls in which predictor variables could not be obtained were excluded from the analysis is a potential source of selection bias, but fortunately the number of such cases and controls were limited. Another potential issue is the choice of control group. Control groups in case-control studies are notoriously difficult to choose, and in studies of CDI, arguments have been made both in favor of, and against, patients with a negative test as a control group [29,30]. A final limitation of the study was that it was powered to study the main hypothesis, but it cannot be excluded that a weak association could go undetected, and it was distinctly underpowered to assess the associations of CDI with individual GABA-modulators.

In conclusion, this study identified no association between the regular use of any included GABA-modulator and the risk of *Clostridium difficile* infection. A trend towards a significant association between the receptor-selective GABA agonist zolpidem and CDI was observed. This association needs to be reassessed in further studies.

## Supporting Information

S1 Table Effect modifiers and interaction variables considered (based on subject matter considerations). (PDF)

S2 Table Predictors and interaction terms included in the propensity score for the propensity to use zolpidem. (PDF)
