# Supplementary material for: The Association between GABA-Modulators and Clostridium difficile Infection – A Matched Retrospective Case-Control Study
Source: PLoS One. 2017 Jan 6;12(1):e0169386. doi: 10.1371/journal.pone.0169386 (PMC5217962; doi:10.1371/journal.pone.0169386)
Supplement: S1 Table — (PDF) [file pone.0169386.s001.pdf]

**S1 Table. Effect modifiers and interaction variables considered (based on subject matter considerations)**

| Variable 1 | Main predictor | *p*-value of interaction term |
|---|---|---|
| **Depression** | Zolpidem use | 0.003* |
| **SSRI use** | Zolpidem use | 0.272 |
| **Benzodiazepine use** | Zolpidem use | 0.697 |
| **Mirtazapin use** | Zolpidem use | 0.298 |
| **Recent hospitalization** | Zolpidem use | 0.435 |
| **Nursing home res.** | Zolpidem use | 0.406 |
| **Variable 1** | **Variable 2** | |
| **Congestive heart failure** | Statin use | 0.554 |
| **Ischemic heart disease** | Statin use | 0.282 |
| **Peptic ulcer disease** | PPI use | 0.583 |
| **Rheumatic disease** | Steroid use | 0.261 |
| **Ulcerous colitis** | Steroid use | 0.188** |
| **Benzodiazepine use** | SSRI use | 0.321 |
| **Depression** | SSRI use | 0.805 |
| **Depression** | Mirtazapin use | 0.200** |
| **Recent hospitalization** | Nursing home residency | 0.321 |
| **Recent hospitalization** | Cephalosporin use | 0.474 |
| **Recent hospitalization** | Clindamycin use | 0.426 |
| **Recent hospitalization** | Penicillin use | 0.721 |
| **Recent hospitalization** | Fluoroquinolone use | 0.290 |
| **Recent hospitalization** | Co-trimoxazole use | 0.218** |
| **Nursing home residency** | Cephalosporine use | 0.019** |
| **Nursing home residency** | Penicillin use | 0.425 |
| **Nursing home residency** | Fluoroquinolone use | 0.698 |
| **Nursing home residency** | Clindamycine use | 0.013** |
| **Nursing home residency** | Co-trimoxazole use | 0.606 |
| **Penicillin use** | Clindamycin use | 0.024** |
| **Penicillin use** | Cephalosporin use | 0.500 |
| **Cephalosporin use** | Clindamycin use | 0.852 |
| **Cephalosporin use** | Fluoroquinolone use | 0.007** |
| **Cephalosporin use** | Co-trimoxazole use | 0.522 |
| **Clindamycin use** | Fluoroquinolone use | 0 |

* Suggests effect modification

** p<0.25, Included in the crude multivariate model
