# Supplementary material for: The Association between GABA-Modulators and Clostridium difficile Infection – A Matched Retrospective Case-Control Study
Source: PLoS One. 2017 Jan 6;12(1):e0169386. doi: 10.1371/journal.pone.0169386 (PMC5217962; doi:10.1371/journal.pone.0169386)
Supplement: S2 Table — (PDF) [file pone.0169386.s002.pdf]

S2 Table. Predictors and interaction terms included in the propensity score for the propensity to use zolpidem

Predictors	Interaction terms
Gender	Congestive heart failure # Statin use
Age	Ischemic heart disease # Statin use
Nursing home residency	Peptic ulcer disease # PPI
Recent hospitalization	Rheumatic disease # Steroid use
Peripheral vascular disease	Ulcerous colitis # Steroid use
History of Myocardial infarction	Benzodiazepine use # SSRI use
Congestive Heart Failure	Depression # SSRI use
Cerebrovascular disease	Depression # Mirtazapin use
Chronic mild liver disease	Recent hospitalization # Nursing home residency
Dementia	Recent hospitalization # Cephalosporine use
Chronic pulmonary disease	Recent hospitalization # Penicillin use
Rheumatologic disease	Recent hospitalization # Clindamycin use
Peptic ulcer	Recent hospitalization # Fluoroquinolone use
Diabetes without organ failure	Recent hospitalization # Co-trimoxazole use
Non-metastatic solid tumor	Nursing home residency # Cephalosporine use
Hemiplegia	Nursing home residency # Penicillin use
Leukemia/Lymphoma	Nursing home residency # Clindamycin use
Diabetes with organ failure	Nursing home residency # Fluoroquinolone use
Chronic kidney disease	Nursing home residency # Co-trimoxazole use
Moderate/severe liver disease	Penicillin use # Cephalosporine use
AIDS	Penicillin use # Clindamycin use
Metastatic malignant tumor	Cefalosporin use # Clindamycin use
Charlson’s Comorbidity index	Cefalosporin use # Fluroquinolone use
Immunodeficiency	Cefalosporin use # Co-trimoxazole use
Chron’s disease	Clindamycin use # Fluoroquinolone use
Ulcerative colitis	
History of depression	
Penicillins	
Cefalosporins	
Clindamycin	
Carbepenems	
Fluoroquinolones	
Tetracycline	
Co-trimoxazole	
SSRI	
Mirtazapin	
PPI	
Statin	
Corticosteroids	
Benzodiazepines	
GABA-analog	
Baclofen	
Zopiclone	
